# The Association of Neuropeptide Y with the Presence and Frequency of Ventricular Premature Beats

**DOI:** 10.2174/011573403X361970250507035931

**Published:** 2025-05-15

**Authors:** Necip Fazıl Dedeoglu, Mustafa Begenc Tascanov, Kenan Toprak, Halil Fedai, Asuman Bicer, İbrahim Halil Altiparmak, Zulkif Tanriverdi, Recep Demirbag, Ismail Koyuncu

**Affiliations:** 1 Department of Cardiology, Faculty of Medicine, Harran University, Sanliurfa, Turkiye;; 2 Department of Biochemistry, Faculty of Medicine, Harran University, Sanliurfa, Turkiye

**Keywords:** Ventricular extra systole, arrhythmia, neuropeptide Y, etiology, cardiology, G protein

## Abstract

**Introduction:**

The estimated prevalence of ventricular extra systole (VES) in the general population is about 1-4% on ECG, but 24-hour Holter monitoring has shown a prevalence of 40-75%. While it may be asymptomatic in many patients, frequent VES persisting for a long time can negatively affect left ventricular (LV) systolic function in patients without structural heart disease. The etiology of VES is not completely known. In this study, we investigated the role of neuropeptide Y (NPY) in the occurrence of VES.

**Materials and Methods:**

In this study, we included 150 patients with VES and 86 cases without VES as the control group. 24-hour Holter monitoring was performed on all subjects. Patients with VES were divided into two subgroups according to the frequency of VES as those >15,000 (Group 1, n= 48) and <15,000 (Group 2, n= 102). Venous blood was collected from all cases for biochemistry parameters and NPY level measurement.

**Results:**

There were statistically significant differences between the two groups in terms of gender, smoking, LVEF, NPY, total cholesterol, LVEDD, SDNN, SDNN INDEX, RMSD, PNN50, LF, HF, VLF, and LF/HF (*p<*0.05, for all). Correlation analysis showed a significant positive correlation between serum NPY level and number of VES (r=0.577, *p=*0.001), LF (r=0140, *p=*0.032), LVEDD (r=0.162, *p=*0.013), and LVESD (r=0.290, *p=*0.001). Conversely, a negative correlation was observed between NPY and RMSSD (r=-0.162, *p=*0.012), SDNN INDEX (r=-0.136, *p=*0.037). Multivariate logistic regression analysis showed that NPY (odds ratio [OR]: 1.204; 95% confidence interval [CI]: 1.103-1.315; *p=*0.001) was an independent predictor of VES development. ROC curve analysis showed that NPY ≥ 47.9 ng/L predicted the occurrence of VES with a sensitivity of 82.0% and specificity of 81.4%. In addition, NPY ≥ 79.8 predicted the frequency of VES with a sensitivity of 85.5% and specificity of 87.3%.

**Conclusion:**

Our study showed that serum NPY levels may play an important role in the development of VES. Also, it was found that the frequency of VES increased as the NPY level increased.

## 1 INTRODUCTION

Ventricular extra systole (VES) occurs in 1-4% of the normal population. Although it may be asymptomatic in many patients, symptoms can develop in others depending on the VES localization and frequency. Studies reported that frequent VES persisting for a long time negatively affect left ventricular (LV) systolic functions in patients without structural heart disease [1]. However, the causes and influencing factors of VES have not been fully identified yet.

Neuropeptide Y (NPY) is a 36-amino acid peptide expressed in all organs of the body, including the airways [2]. It binds to a series of G protein-coupled receptors (GPCRs) belonging to the rhodopsin-like superfamily (class 1) [3], and plays a role in respiratory, cardiovascular, and endocrine systems, as well as in neuronal excitability, depression, anxiety, neuroendocrine secretion, and vasoconstriction [4-6]. NPY is also widely found in both the central and peripheral nervous systems and plays a role in physiological processes such as sympathetic activation [7]. Moreover, NPY is commonly found in peripheral and central neurons [8]. In the peripheral neurons, it is found in nerve plexuses around the blood vessels of other organs, in adrenergic nerve terminals, and the adrenal medulla, where it coexists and is co-released with norepinephrine [9]. Although NPY is generally found in sympathetic nerves, it has sometimes been shown to be present in parasympathetic nerves [10].

In the cardiovascular system, it is found in neurons supplying blood vessels, myocytes, and the endocardium. It is involved in some processes such as vasoconstriction, cardiac remodeling, and angiogenesis [11, 12]. NPY has become increasingly prominent in the pathogenesis of cardiovascular diseases, including hypertension (HT), atherosclerosis, acute myocardial infarction (AMI), arrhythmia, and heart failure (HF) [13].

Arrhythmias are defined as any pathological conditions affecting the heart's electrical activity [14], and VES is one of them. Symptoms of VES may include palpitations and chest pain, and they can progress to ventricular tachycardia, causing hypotension, collapse, and acute heart failure [15]. Although the clinical causes of VES are attributed to specific reasons, their molecular causes have not been fully elucidated yet. The reasons for significant symptomatic differences among individuals and the events caused by these differences have not been fully clarified. In this study, we aimed to investigate the possible effect of NPY on the development of VES.

## 2 MATERIALS AND METHODS

### 2.1 Study Population

The study was designed prospectively. A total of 236 consecutive patients who visited the Cardiology Clinic of Harran University Medical Faculty between 2022 and 2023 and had indications for a 24-hour rhythm Holter monitoring were investigated. 150 individuals with ventricular premature beats and 86 individuals without VES were included in the study, and comparisons were made. Also, because previous studies demonstrated that individuals with VES over 15,000 have been shown to have an increased risk of cardiac events, our VES group were divided into two groups to reveal the clinical significance of NPY on VES frequency (those with more than 15,000 VES; Group 1 and those with fewer than 15,000 VES; Group 2) [16, 17].

All patients underwent detailed physical examinations following the collection of their medical histories. Risk factors such as HT, diabetes mellitus (DM), hyperlipidemia, and smoking were recorded. Hypertension was defined as systolic blood pressure of 140 mmHg or higher and/or diastolic blood pressure of 90 mmHg or higher. Hyperlipidemia was defined as an LDL cholesterol level of 130 mg/dL or higher, a triglyceride level of 150 mg/dL or higher, and a total cholesterol level of 200 mg/dL or higher. Diabetes Mellitus (DM) was defined as a fasting blood glucose level of 126 mg/dL or higher and a postprandial blood glucose level of 200 mg/dL or higher, resulting from abnormalities in insulin secretion, insulin action, or both.

Electrocardiography (ECG) and echocardiography were performed for all subjects, and blood samples were collected for routine laboratory tests. Patients with structural heart disease (known coronary artery disease, heart failure, valve diseases, *etc*.), electrolyte imbalances, arrhythmias other than VES on ECG or 24-hour rhythm Holter, antiarrhythmic drug usage, signs of acute infection, those under the age of 18, and those with chest pain and shortness of breath who were evaluated to exclude possible coronary artery disease were not included in the study. The study was approved by the Ethics Committee of Harran University Medical Faculty (number: HRU/21.12.18, date 21.06.2021) and was conducted under the Helsinki Declaration. Informed consent was obtained from all patients.

### 2.2 Biochemical Analysis and Serum Neuropeptide Y Test

Venous blood was collected into a standard biochemistry tube, and standard laboratory methods were used to analyze blood glucose, electrolytes, total cholesterol, high-density lipoprotein, low-density lipoprotein, and triglyceride levels. Additionally, one tube of blood was collected for the NPY test. The blood samples were centrifuged at 3000 rpm for 10 minutes, and the serum was stored at -80°C until the day of analysis. NPY levels were measured using an enzyme-linked immunosorbent assay according to the manufacturer's instructions (Sunred, Shanghai, China).

#### 2.2.1 Rhythm Holter ECG

A 24-hour rhythm Holter ECG (Bi Biomed DMS Cardioscan, USA) was recorded for all patients. The number of VESs, SDNN, SDNN INDEX, RMSSD, PNN50, HF, LF, VLF, LF/HF, standard QRS, and average QRS were automatically calculated from the recordings in the 24-hour rhythm Holter ECG.

Heart rate variability (HRV) in the 24-hour Holter is a parameter that helps to explain the effects of the autonomic nervous system on the cardiac system. The autonomic nervous system causes and maintains ventricular arrhythmias in the cardiac system. HRV is obtained from the R-R intervals of normal cardiac beats in the Holter. It is divided into time-dependent and frequency-dependent parameters.

Time-dependent parameters: SDNN, SDNN INDEX, RMSSD, and PNN50, which indicate parasympathetic activation.

SDNN: Indicates the standard deviation of the differences between all NN intervals throughout the examination. It shows parasympathetic activity. A low value is significant, whereas a high value does not carry a negative meaning. A low value suggests rhythm disturbance. Studies have found that an SDNN > 100 ms results in an annual mortality of approximately 5.5%, while an SDNN < 50 ms results in an annual mortality of approximately 51.4% in chronic heart failure patients [18].

SDNN INDEX: It shows the standard deviation of average NN intervals for each 5 min segment of a 24-hour Holter recording [19].

PNN50 (Percentage of RR): The percentage of the difference between NN intervals.

RMSSD (Root mean square of the RR interval difference): The root mean square of the sum of the squares of differences in NN intervals in a 24-hour recording.

Frequency-dependent parameters: HF, LF, VLF, and LF/HF indicate sympathetic and parasympathetic activation.

Very low frequency (VLF) power: Shows the magnitude of oscillation in the heart rate model at different frequencies and speeds in 15 seconds and every 5 minutes. It provides information about the parasympathetic system [20].

Low-frequency (LF) power: Indicates the magnitude of oscillation in heart rate within the range of three to nine cycles per minute. It shows both parasympathetic and sympathetic systems in a combined manner, with the sympathetic system being more dominant [21].

High-frequency (HF) power: Indicates heart rate oscillations within the range of nine to 24 cycles per minute and is typical of adult respiratory frequency ranges. It shows parasympathetic activity [22].

LF/HF: Indicates the sympathovagal balance. An increased ratio shows sympathetic dominance. While LF can show both parasympathetic and sympathetic balance, HF only indicates the parasympathetic system [20, 21].

### 2.3 Statistical Analysis

Statistical analyses were performed using IBM SPSS Statistics software (version 22.0; IBM Corp., Armonk, NY, USA). The Kolmogorov-Smirnov test was used to determine whether continuous variables were distributed normally. Continuous variables were expressed as mean ± standard deviation (SD) or median (interquartile range), depending on the distribution. The Student's t-test or Mann-Whitney U test was used for numerical variables, and the chi-square (χ2) test was used for categorical variable analyses. Pearson or Spearman correlation coefficient was used to determine the correlation of NPY with other continuous variables. Univariate logistic regression analysis was performed to determine the possible causes of VES formation and frequency, and multivariate logistic regression analysis was used to identify independent predictors. ROC curve analysis was performed to determine the optimal cut-off value of NPY for predicting VES formation and frequency. A *p*-value of <0.05 was considered statistically significant.

## 3 RESULTS

A total of 150 patients who detected VES in the 24-hour Holter recording (mean age: 47.93 ± 13 years, 54% male) and 86 patients without any VES (mean age: 44.81 ± 13 years, 60% male) were included in this study. No statistical difference was found between the two groups regarding age, body mass index (BMI), diabetes mellitus (DM), blood pressure, heart rate, hemoglobin (HGB), LDL, triglycerides (TG), white blood cell count (WBC), creatinine, urea, Na, K, CRP, platelets (PLT), TSH, IVS, LVEDV, PWD, STANDARD QRS, and MEAN QRS (*p* > 0.05) (Table **1**). However, gender (*p =* 0.032), LVEF (0.001), NPY (*p <* 0.001), total cholesterol (*p =* 0.020), SDNN (*p =* 0.001), SDNN INDEX (*p =* 0.014), RMSSD (*p =* 0.036), PN50 (*p =* 0.025), LF (*p =* 0.017), HF (*p =* 0.003), VLF (*p =* 0.011), and LF/HF (*p =* 0.001) were higher in the VES group compared to the non-VES group (Tables **1** and **2**).

The VES group was also divided into two groups to reveal the clinical importance of NPY on VES frequency as follows: VES frequency ≥15,000 (GROUP 1, n = 48, 32%) and <15,000 (GROUP 2, n = 102, 68%). The two groups were compared based on NPY levels and Holter parameters.

Patients in group 1 had significantly higher NPY [(80 (63-113) *vs*. 70 (50-82), *p =* 0.007)], LF [(529 (311-1010) *vs*. 411 (219-707), *p =* 0.012)], HF [(204 (98-431) *vs*. 142 (79-274), *p =* 0.065)], and LF/HF [(2.4 (1.5-4) *vs*. 2.4 (1.5-3.8), *p =* 0.926)], whereas lower VLF [(613 (433-941) vs. 779 (510-1122), *p =* 0.036)], SDNN [105 (82-139) *vs*. (117 (91-147), *p =* 0.023)], SDNN INDEX [49.5 (34.5-65.75) *vs.* 54.5 (41.75-72.5), *p =* 0.016)], RMSSD [24 (14-32) *vs*. (29 (21-42), *p =* 0.001)], and PNN50 [5 (1-10) *vs*. (7 (3-17), *p =* 0.015)] (Table **3**).

In correlation analysis, NPY was positively correlated with VES count (r = 0.577, *p =* 0.001), LF (r = 0.140, *p =* 0.032), and LVESD (r = 0.290, *p =* 0.001). Conversely, NPY was negatively correlated with RMSSD (r = -0.162, *p =* 0.012) and SDNN INDEX (r = -0.136, *p =* 0.037) (Table **4**). In addition, VES count was positively correlated with LF/HF (r = 0.234, *p <* 0.001), and LF (r = 0.242, *p <* 0.001); whereas negatively correlated with EF (r = -0.234, *p <* 0.001), VLF (r = -0.252, *p <* 0.001), SDNN (r = -0.228, *p <* 0.001), SDNNINDEX (r = -0.226, *p <* 0.001), RMSSD (r = -0.258, *p <* 0.001), and PNN50 (r = -0.191, *p <* 0.003) (Table **5**).

Univariate logistic regression analysis revealed that age [(1.046 (1.018 – 1.065) *p<*0.001)], gender [(0.557 (0.315 – 0.954) *p=*0.033)], SDNN [(0.988 (0.981 – 0.994) *p<*0.001)], SDNNİNDEX [(0.981 (0.971 – 0.993) *p=*0.001)], PNN50 [(0.969 (0.946 – 0.991) *p=*0.008)], HF [(0.999 (0.999 – 1.000) *p=*0.019)], VLF [(0.999 (0.998 – 1.000) *p=*0.007)] LF [(1.001 (1.000 – 1.001) *p=*0.001)], EF [(0.909(0.857 – 0.965) *p<*0.001)], NPY [(1.010 (1.004 – 1.017) *p=*0.003)] It revealed that there are possible factors responsible for the formation of VES.

Multivariate logistic regression analysis showed that age (odds ratio [OR], 1.060; 95% confidence interval [CI], 1.031–1.088; *p <*0.001), serum NPY (OR, 1.008; 95% CI, 1.001–1.015; *p =* 0.010), and LF (OR, 1.004; 95% CI, 1.001–1.006; *p <*0.001) were independent predictors of VES occurrence (Table **6**).

**Fig. (1):**
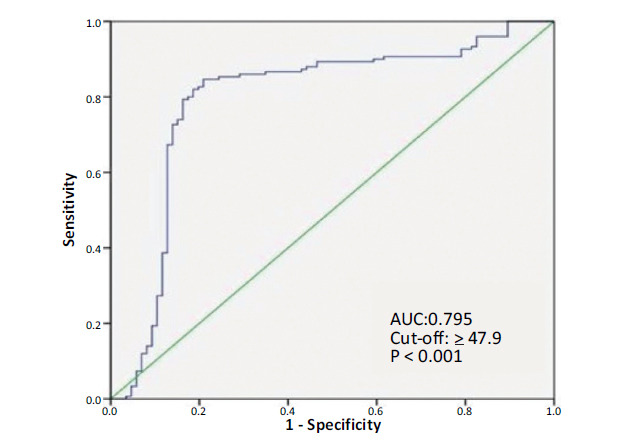
Receiver operating characteristic (ROC) curve analysis of NPY for predicting VES formation.

ROC curve analysis was used to determine the optimal cut-off value of serum NPY to predict VES formation. NPY ≥ 47.9 ng/L predicted VES formation with a sensitivity of 82.0% and a specificity of 81.4% (area under the curve: 0.795, 95% CI: 0.717–0.861, *p <* 0.001) (Fig. **1**).

**Fig. (2):**
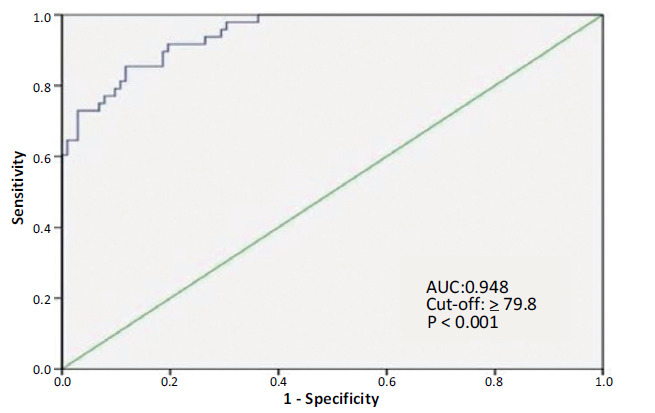
Receiver operating characteristic (ROC) curve analysis of NPY for predicting VES frequency.

Univariate logistic regression analysis revealed that RMSSD [(0.927 (0.892 – 0.963), *p <* 0.001)], SDNN [(0.988 (0.979 – 0.997), *p =* 0.007)], SDNNINDEX [(0.973 (0.955 – 0.991), *p =* 0.003)], PNN50 [(0.933 (0.887 – 0.981), *p =* 0.007)], HF [(1.001 (1.000 – 1.002), *p =* 0.032)], VLF [(0.999 (0.998 – 1.000), *p =* 0.021)], LF [(1.001 (1.000 – 1.002), *p =* 0.008)] and NPY [(1.146 (1.082 – 1.215), *p <* 0.001)] could be responsible for VES frequency. Multivariate logistic regression analysis indicated that PNN50 (odds ratio [OR], 0.798; 95% confidence interval [CI], 0.671–0.948; *p =* 0.010), serum NPY levels (OR, 1.204; 95% CI, 1.103–1.315; *p =* 0.001), and VLF (OR, 0.997; 95% CI, 0.995–1.000; *p =* 0.027) were the independent predictors of VES frequency (Table **7**). ROC curve analysis was used to determine the optimal cut-off value of NPY for predicting VES frequency. NPY ≥ 79.8 ng/L predicted VES frequency with 85.4% sensitivity and 87.3% specificity (area under the curve: 0.948, 95% CI: 0.915–0.980, *p <* 0.001) (Fig. **2**).

## 4 DISCUSSION

In this study, NPY levels were examined in patients with and without VES who underwent 24-hour rhythm Holter monitoring. The significant findings are as follows: (1) NPY levels were significantly higher in patients with VES compared to those without VES; (2) Serum NPY levels significantly increased with the frequency of VES; (3) NPY was an independent predictor of VES development; (4) Parameters indicating the autonomic nervous system, such as LF, HF, and LF/HF, were significantly different between the groups.

The autonomic nervous system can play an important role in triggering and sustaining cardiac arrhythmias. Heart rate variability (HRV) analyses through the autonomic nervous system can be used to explain these situations. In some cases, arrhythmias may be triggered by the activation of the sympathetic nervous system. The parasympathetic system reduces heart rate and suppresses the sympathetic system. Cardiac arrhythmias are categorized into atrial and ventricular groups. VES is included in the ventricular group and are premature depolarizations originating from the ventricles. The estimated prevalence in the general population is 1-4% on ECG, but 24-hour Holter monitoring has shown a prevalence of 40-75% [23, 24]. Although VES is considered benign, it has been associated with unwanted cardiac events such as heart failure and sudden death. The mechanism of VES development is known, but its etiology includes activation of the sympathetic nervous system, electrolyte imbalances, myocardial ischemia, and structural heart diseases. Additionally, many biochemical parameters have been evaluated to show the etiology of VES [25]. NPY is one of the parameters evaluated for VT etiology following myocardial ischemia [26]. NPY is a sympathetic co-transmitter widely found in the peripheral and central nervous systems and has physiological effects. It is the most abundant neuropeptide in the heart and is also present in the vascular system, postganglionic sympathetic neurons supplying cardiomyocytes, as well as in intracardiac ganglia and parasympathetic neurons [27]. NPY has six different G-protein-coupled receptors. The Y1 receptor plays a role in most peripheral effects and is usually found on postsynaptic membranes. This receptor exerts its inhibitory effects on adenylate cyclase in vascular smooth muscle. In addition, Y1 receptor blockade has been shown to have protective effects against adverse cardiac remodeling in mouse cardiac myocytes, and therefore, Y1 receptor antagonism may have therapeutic potential in heart diseases [11-28]. The Y2 receptor is found in areas of the brain such as the thalamus, hypothalamus, and brainstem. It is also located in sensory, parasympathetic, and sympathetic neurons in the peripheral nervous system [9]. The Y3 receptor is a receptor for which sufficient data is lacking, although it has been cloned in mice [28]. The Y4 receptor is highly specific for pancreatic polypeptide (PP) and is thought to be the intracellular receptor of PP. In humans, it is found in the large intestine, pancreas, and small intestine [3]. The Y5 receptor is highly concentrated in the testes and brain of mice, with a similar distribution in humans. It is also located in regions such as the hypothalamus and amygdala, where emotional behavior is regulated [9, 29]. The Y6 receptor is incomplete and is abundant in the heart. It has also been shown to be present in the skeletal system, gastrointestinal system, and adrenal gland. However, its role in human physiology has not yet been determined [30].

NPY is involved in physiological processes such as cardiac remodeling and angiogenesis and plays a role in the pathogenesis of cardiac diseases like arrhythmia, atherosclerosis, and heart failure [27]. In our study, we found that serum NPY levels were significantly higher in the VES group compared to the control group. We also found a strong positive correlation between VES and NPY. Additionally, serum NPY levels were significantly higher in patients with VES ≥ 15,000 compared to those with VES < 15,000. We demonstrated that NPY was an independent predictor of VES development and frequency. Our results suggest that NPY may be used to detect the presence and frequency of VES. The 24-hour rhythm Holter is an easy and non-invasive tool used for diagnosing and monitoring cardiac arrhythmias. HRV parameters from Holter recordings are used to evaluate the sympathetic and parasympathetic nervous systems responsible for cardiac arrhythmia pathogenesis. Previous studies have shown that changes in HRV parameters are associated with adverse cardiac events [31].

In our study, indirect activation of the sympathetic nervous system, indicated by LF and LF/HF values, was found to be significantly higher in the group without VES. Additionally, a weak correlation was observed between VES and LF/HF, LF. LF/HF indicates the sympathovagal balance. As the ratio increases, sympathetic dominance increases. Furthermore, LF was identified as an independent predictor of VES formation. These results indicate that sympathetic nervous system activity is increased in VES formation. Additionally, parameters indicating indirect activation of the parasympathetic nervous system, such as SDNN, SDNNINDEX, RMSSD, and PNN50, were significantly lower in the VES group. These parameters showed a negative correlation with VES, suggesting suppression of the parasympathetic nervous system.

NPY, LF, HF, LF/HF, VLF, SDNN, SDNNINDEX, RMSSD, and PNN50 values were significantly different between patients with VES ≥ 15,000 and <15,000. In multivariate logistic regression analysis, PNN50 and VLF were the independent predictors of VES frequency. These findings suggest that the increased sympathetic activity may be due to compensatory activation of the parasympathetic nervous system. We think that larger prospective studies are needed to better elucidate this relationship. This finding in our study may indicate increased sympathetic activity. We also observed significantly higher VES frequency in smokers. However, the effect of smoking on NPY is not known. More extensive prospective studies are needed to better determine the relationship between NPY and VES development. Consequently, when VES is detected in patients presenting with palpitations, the presence and frequency of VES can be estimated according to the NPY serum level. In addition, the relationship of NPY with LF, HF, and LF/HF parameters may help us understand the role of sympathovagal imbalance in the development of VES. This may shed light on new advances and approaches for stress and autonomic dysfunction-induced arrhythmias.

## 5 STUDY LIMITATIONS

The relatively small sample size and single-center nature of the study limit its significance. Also, our study examined only the presence or absence of VES in the 24-hour rhythm Holter and excluded other arrhythmias. Comparison with other arrhythmias and long-term follow-up of patients could have provided additional value to our study. Furthermore, our study was a cross-sectional study, and follow-up data were not recorded. Further multicenter and larger prospective studies are needed to better clarify the relationship between NPY and VES development.

## CONCLUSION

In this study, NPY levels were examined in patients with and without VES who underwent 24-hour rhythm Holter monitoring. NPY levels were significantly higher in patients with VES compared to those without VES. In addition, serum NPY levels significantly increased with VES frequency. Furthermore, NPY was an independent predictor of VES development. Parameters indicating the autonomic nervous system, such as LF, HF, and LF/HF, were significantly different in the group with VES.

## Figures and Tables

**Table 1:** Basic demographic, clinical and laboratory characteristics of the study groups.

**Variables**	**Patient Group (n:150)**	**Control Group (n:86)**	***P-value***
Age (years)	47.93 ± 13	44.81 ± 13	0.113
Gender Male n, (%) Female n, (%)	81 (54.00) 69 (46.00)	52 (60.50) 34 (39.50)	0.032
Smoking	49 (0.32)	15 (0.17)	0.015
BMI (kg/m^2^)	26.48 ± 0.20	26.17 ± 0.33	0.403
Systolic Blood pressure (mmHg)	115.4 ± 1.70	119.7 ± 1.50	0.076
Diastolic Blood pressure (mmHg)	70.80 ± 0.88	69.90 ± 1.10	0.569
Diabetes mellitus%)	25 (16.66)	7(8.13)	0.076
Total cholesterol (mg/dL)	174.9 ± 3.09	162.7 ± 4.27	0.020
HDL (mg/dl)	38.2 ± 0.91	42.1 ± 1.73	0.103
LDL (mg/dl)	103.9 ± 2.34	100 ± 3.34	0.449
TG (mg/dl)	183.7 ± 8.15	163.9 ± 12.80	0.172
HGB (g/dl)	13.9 ± 0.19	13.8 ± 2.17	0.915
Creatinine (mg/dl)	0.92 ± 0.65	0.78 ± 0.03	0.128
Urea (mg/dl)	29 ± 1.26	26 ± 1.31	0.070
WBC (10e3/ul)	9.3 ± 0.70	8.0 ± 0.29	0.166
NPY (ng / L)	73 ± 4.31	35 ± 7.77	<0.001
K (mmol/L)	4.3 ± 0.03	4.3 ± 0.04	0.443
Na (mmol/L)	140.8 ± 0.30	140.8 ± 0.20	0.028
Plt (10e3/ul)	271.3 ± 6.50	281.8 ± 8.10	0.323
hsCRP (mg/dL)	0.83 ± 0.12	0.75 ± 0.06	0.652
TSH (mIU/L)	1.64 ± 0.08	1.66 ± 0.10	0.838
LVEF (%)	56.1 ± 4.92	58.7 ± 2.90	<0.001
IVS (cm)	1.04 ± 0.07	0.92 ± 0.02	0.254
PWD (cm)	1.2 ± 0.06	0.7 ± 0.40	0.357
LVEDD (cm)	4.90 ± 0.47	4.72 ± 0.42	0.489

**Abbreviations:** BMI: Body mass index, HDL: High dansity protein, LDL: Low dansity proein, TG: Trigliserid, NPY: Neuropeptide Y, IVS: Interventricular septum, LVEF: Left ventricular ejection fraction, PWD: Posterior wall thickness, LVEDD: Left ventricular end-diastolic diameter, Plt: Platelet, TSH: Thyroid stimulating hormone, HGB: Hemoglobin, K: Potassium, Na: sodium, WBC: White blood cell, hsCRP: High sensitive C reaktive protein.

**Table 2:** Characteristics of the study groups according to Holter parameters.

**Variables**	**Patient Group (n:150)**	**Control Group (n:86)**	***P-value***
Heart rate (min/per)	78.1 ± 10.1	75.2 ± 13.3	0.162
SDNN (ms)	115 (88-143)	145 (108-178)	<0.001
SDNN INDEX (ms)	53 (39-68)	58 (47-77)	0.014
RMSSD (uv)	27 (20-36)	29 (23-44)	0.036
PNN50 (%)	6 (2-13)	8 (4-20)	0.027
HF (Herz)	164 (83-298)	241 (112-548)	0.003
LF(Herz)	467 (244-768)	409 (162-607)	0.017
VLF (Herz)	735 (500- 1030)	847 (636-1117)	0.011
LF/HF(Herz)	2.4 (1.5-4)	1.7 (0.9-2.9)	<0.001
Standard QRS (ms)	129 ± 1.8	128 ± 2.3	0.581
Mean QRS (ms)	113 ± 2	112 ± 3	0.907

**Abbreviations:** SDNN: Standard deviation of RR intervals, SDNN INDEX: Standard deviation of RR intervals in 5 min, HF: High frequency, LF: Low frequency, VLF: Very low frequency, PNN50: Ratio of the number of neighboring NN intervals to the total number of NN intervals, RMSSD: Root mean square of the differences of neighboring RR intervals.

**Table 3:** Comparison of parameters according to VES frequency.

**Variables**	**Group 1**	**Group 2**	***P-value***
NPY (ng / L)	113 (84-185)	64 (46-75)	<0.001
LF (Herz)	529 (311-1010)	411 (218-707)	0.012
HF (Herz)	204 (98-431)	142 (79-274)	0.065
LF/HF (Herz)	2.5 (1.6-4.0)	2.4 (1.5-3.8)	0.926
VLF (Herz)	613 (433-941)	779 (510-1122)	0.036
SDNN (ms)	105 (82-139)	117 (91-147)	0.023
SDNN INDEX (ms)	49.5 (34.5-65.75)	54.5 (41.75-72.5)	0.016
RMSSD (uv)	24 (14-32)	29 (21-42)	< 0.001
PNN50 (%)	5 (1-10)	7 (3-17)	0.015

**Note: Group 1:** Number of VES≥15000, **Group 2**: Number of VES<15000 SDNN.

**Abbreviations:** Standard deviation of RR intervals, SDNN INDEX: Standard deviation of RR intervals in 5 min, HF: High frequency, LF: Low frequency, VLF: Very low frequency, PNN50: Ratio of the number of neighboring NN intervals to the total number of NN intervals, RMSSD: Root mean square of the differences of neighboring RR intervals, NPY: Neuropeptide Y.

**Table 4:** Correlation analysis according to Neuropeptide Y.

**Variables**	**r**	***P-value***
Age (year)	-0.054	0.409
Heart rate (min/per)	-0.149	0.022
VES (count/day)	0.456	<0.001
LVEDD (cm)	0.480	0.489
LF (Herz)	0.140	0.032
RMSSD (uv)	-0.162	0.012
SDNN INDEX (ms)	-0.136	0.037

**Abbreviations:** SDNNINDEX: Standard deviation of RR intervals in 5 min, LF: Low frequency, RMSSD: Root mean square of the differences of adjacent RR intervals, VES: Ventricular extrasystole, LVEDD: Left ventricular end-diastolic diameter.

**Table 5:** Correlation analysis between VES number and clinical laboratory, echocardiographic and Holter parameters.

**Variables**	**r**	***P-value***
Age (year)	0.083	0.204
LVEDD (cm)	0.253	0.489
LF/HF (Herz)	0.234	<0.001
LF (Herz)	0.242	<0.001
NPY (ng / L)	0.456	<0.001
LVEF (%)	-0.234	<0.001
VLF (Herz)	-0.252	<0.001
SDNN (ms)	-0.228	<0.001
SDNN INDEX (ms)	-0.226	<0.001
RMSSD (uv)	-0.258	<0.001
PNN50 (%)	-0.191	<0.003

**Abbreviations:** SDNN: Standard deviation of RR intervals, SDNNINDEX: Standard deviation of RR intervals in 5 min, LF: Low frequency, VLF: Very low frequency, PNN50: Ratio of adjacent NN interval number to total NN interval number, RMSSD: Root mean square of the differences of adjacent RR intervals, NPY: Neuropeptide y, LVEDD: Left ventricular end-diastolic diameter, LVEF: Left ventricular ejection fraction.

**Table 6:** Univariate and multivariate logistic regression analysis representing independent determinants of VES formation.

**Variables**	**Odds Ratio (95%CI)**	***P-value***	**Odds Ratio (95% CI)**	***P-value***
Age	1.805 (0.436-6.675)	0.113	1.014 (0.990-1.040)	0.260
Gender	0.557 (0.325-0.954)	0.033	0.710 (0.958-3.614)	0.067
BMI	1.042 (0.946-1.149)	0.402	-	-
LVEDD	2.389 (1.358-4.201)	0.003	1.105 (0.528-2.311)	0.792
LVEF	0.909 (0.857-0.965)	0.002	0.975 (0.894-1.064)	0.568
RMSSD	972 (0.966-0.990)	0.742	1.002 (0.968-1.037)	0.908
SDNN	0.988 (0.982-0.994)	< 0.001	0.989 (0.980-0.997)	0.010
SDNN INDEX	0.982 (0.971-0.993)	0.002	0.997 (0.980-.015)	0.756
PNN50	0.969 (0.946-0.992)	0.008	1.006 (0.963-1.051)	0.786
HF	0.999 (0.999-1.000)	0.029	0.999 (0.997-1.000)	0.036
VLF	0.999 (0.998-1.000)	0.007	0.999 (0.998-1.000)	0.088
LF	1.001 (1.000-1.002)	0.002	1.002 (1.001-1.004)	0.001
LF/HF	1.079 (0.984-1.184)	0.104	-	-
NPY	1.010 (1.004-1.017)	0.003	1.009 (1.002-1.016)	0.009

**Abbreviations:** SDNN: Standard deviation of RR intervals, SDNN INDEX: Standard deviation of RR intervals in 5 min, HF: High frequency, LF: Low frequency, VLF: Very low frequency, PNN50: Ratio of adjacent NN intervals to total NN intervals, RMSSD: Root mean square of differences of adjacent RR intervals, NPY: Neuropeptide Y, LVEDD: Left ventricular end-diastolic diameter, LVEF: Left ventricular ejection fraction, BMI: Body mass index, CI: Confidense interval.

**Table 7:** Univariate and multivariate logistic regression analysis representing independent determinants of VES frequency.

**Variables**	**Odds Ratio (95% CI)**	***P-value***	**Odds Ratio (95% CI)**	***P-value***
Age	1.005 (0.984-1.028)	0.626	-	-
Gender	0.600 (0.298-1.210)	0.154	-	-
BMI	1.026 (0.895-1.176)	0.715	-	-
RMSSD	0.927 (0.892-0.963)	<0.001	0.946 (0.858-1.044)	0.273
SDNN	0.988 (0.979-0.997)	0.007	0.982 (0.953-1.011)	0.215
SDNN INDEX	0.973 (0.955-0.991)	0.003	0.959 (0.905-1.016)	0.156
PNN50	0.933 (0.887-0.981)	0.007	0.798 (0.671-0.948)	0.010
HF	1.001 (1.000-1.002)	0.032	1.004 (1.000-1.008)	0.056
VLF	0.999 (0.998-1.000)	0.021	0.997 (0.995-1.000)	0.027
LF	1.001 (1.000-1.002)	0.008	1.002 (1.000-1.005)	0.070
LF/HF	1.006 (0.996-1.016)	0.226	-	-
LVEF	0.992 (0.957-1.028)	0.658	-	-
LVEDD	1.788 (0.951-3.361)	0.071	-	-
NPY	1.146 (1.082-1.215)	<0.001	1.204 (1.103-1.315)	0.001

**Abbreviations:** SDNN: Standard deviation of RR intervals, SDNN INDEX: Standard deviation of RR intervals in 5 min, HF: High frequency, LF: Low frequency, VLF: Very low frequency, PNN50: Ratio of adjacent NN intervals to total NN intervals, RMSSD: Root mean square of differences of adjacent RR intervals, NPY: Neuropeptide Y, LVEDD: Left ventricular end-diastolic diameter, LVEF: Left ventricular ejection fraction, BMI: Body mass index.

## Data Availability

The authors confirm that the data supporting the fndings of this research are available within the article.

## LIST OF ABBREVIATIONS

BMI: Body Mass Index
DM: Diabetes Mellitus
ECG: Electrocardiography
HRV: Heart Rate Variability
LV: Left Ventricular
NPY: Neuropeptide Y
VES: Ventricular Extra Systole

## STANDARDS OF REPORTING

STROBE guidelines were followed.
